# The use of ultrasound-estimated bladder weight in diagnosing bladder outlet obstruction and detrusor overactivity in men with lower urinary tract symptoms

**DOI:** 10.4103/0970-1591.45547

**Published:** 2009

**Authors:** Fadi Housami, Marcus Drake, Paul Abrams

**Affiliations:** Bristol Urological Institute, Bristol, UK

**Keywords:** Bladder outlet obstruction, bladder wall thickness, bladder weight, detrusor overactivity

## Abstract

**Objectives:**

Measurement of bladder weight using ultrasound estimates of bladder wall thickness and bladder volume is an emerging clinical measurement technique that may have a role in the diagnosis of lower urinary tract dysfunction. We have reviewed available literature on this technique to assess current clinical status.

**Methods:**

A systematic literature search was carried out within PubMed and MedLine to identify relevant publications. These were then screened for relevance. Preliminary results from our clinical experiments using the technique are also included.

**Results:**

We identified 17 published papers concerning the technique which covered clinical studies relating ultrasound-estimated bladder wall thickness to urodynamic diagnosis in men, women, and children together with change in response to treatment of bladder outlet obstruction. The original manual technique has been challenged by a commercially available automated technique.

**Conclusion:**

Ultrasound-estimated bladder weight is a promising non-invasive technique for the categorization of storage and voiding disorders in both men and women. Further studies are needed to validate the technique and assess accuracy of diagnosis.

## INTRODUCTION

Lower urinary tract symptoms (LUTS) have an increasing prevalence in ageing men and women. Men who present with LUTS are investigated for benign prostatic enlargement (BPE) and bladder outlet obstruction (BOO), both of which are usually the result of benign prostatic hyperplasia (BPH).[1] However, LUTS alone are not sufficient in diagnosing BPE or BOO and other investigations are usually required.[2]

Uroflowmetry is a cheap test which provides some information on the voiding function and is easy to perform in the clinic setting. However, it lacks the required specificity as it is unable to differentiate between bladder outlet obstruction and detrusor underactivity. Conversely, pressure-flow studies remain the reference test in diagnosing BOO as they are able to provide valuable information on the detrusor contractility as well as the presence or absence of obstruction. This, unfortunately, does not come cheaply as urodynamics are invasive tests and require specialist equipment and training to perform the tests and interpret the results.[3]

## BLADDER WALL THICKNESS AND BLADDER WEIGHT

The quest for a non-invasive test diagnostic of BOO has been ongoing for many years. Many parameters were investigated including free uroflowmetry, post-void residual volume and quantification of prostate volume.[4] Over the past decade or so, interest into bladder wall thickness and consequently bladder wall weight has grown rapidly. This was based on the rationale that bladder outlet obstruction is associated with detrusor hypertrophy and an increase in bladder wall thickness. In fact, morphological studies showed that the increase in bladder wall thickness was the result of smooth muscle hypertrophy as well as increased collagen deposition in the bladder wall.[5]

**Figure 1:**
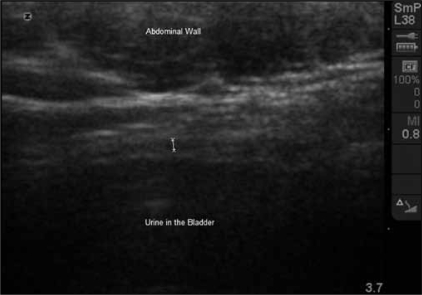
Trans-abdominal ultrasound image of the bladder at 3.7 MHz showing the abdominal wall and bladder. The detrusor is identified as a hypoechoic region sandwiched between an inner and outer hyperechoic layer characteristic of the mucosa and adventitia, respectively

Ultrasound emerged as the easiest and least invasive option in measuring bladder wall thickness. The bladder wall appears on ultrasound as a three layer structure with the detrusor muscle represented by a hypoechogenic layer between two hyperechogenic layers representing the serosa and mucosa [Figure 1].[6] Some investigators measured the thickness of the three layers together,[7] whilst others used the middle detrusor layer only.[8] Most studies used the anterior bladder wall, however, some used the posterior bladder wall as transrectal or transvaginal ultrasound was used.[9] Studies have shown that there are no significant differences in the thickness of the various parts of the bladder wall.[10,11]

Ultrasound imaging is dependent on the frequency of the ultrasound waves; the higher the frequency, the better the resolution of the image but the lower the depth of penetration.[12] Oelke *et al*. suggested that it is necessary to use high-frequency ultrasound arrays (7.5 MHz or higher) with an enlargement function of the ultrasound picture for precise measurement of detrusor wall thickness (DWT).[8]

The problem with bladder wall thickness is that it is volume dependent; wall thickness decrease with increasing filling volume. Oelke *et al*. studied 9 volunteers with normal urodynamics and found that DWT decreased rapidly during the first 250 ml of bladder filling.[8] This prompted others to investigate bladder wall weight as a measure of bladder hypertrophy which should remain constant at different bladder volumes.[10]

## METHODS (1)

### Ultrasound-estimated bladder weight

We reviewed the published literature relating to ultrasound-estimated bladder weight using the search string below and then cross-referencing from the results of the search:

(Title:(bladder) OR (detrusor)) AND (Title:(weight)) AND ((ultraso*) OR (sonogr*))

This search returned 18 articles, one of which was not relevant to the topic. Using “mass” as an alternative to “weight” in the search terms did not add any further relevant articles.

### Results (1)

Almost half of the 17 articles were from a group based at Kyoto Prefectual University of Medicine in Japan. Kojima and associates investigated ultrasound-estimated bladder weight and its relationship with various lower urinary tract dysfunctions in adults as well as children.

Kojima and associates measured bladder wall thickness using a high frequency 7.5 MHz probe at the anterior bladder wall. Intravesical volume was measured by catheterization after measuring bladder wall thickness and then the following steps were used to calculate the ultrasound-estimated bladder weight:[13]

1. Using the known intravesical volume and assuming that the bladder is a sphere it is possible to calculate the internal radius of the bladder.
2. Bladder wall thickness is added to the internal radius to estimate the outer radius.
3. Using the outer radius, it is possible to calculate the total vesical volume.
4. Bladder wall volume is calculated as the difference between the total vesical volume and the intra-vesical volume.
5. Finally ultrasound-estimated bladder weight is calculated by multiplying the bladder wall volume with the specific gravity of the bladder tissue.

In studies on 19 bladders excised at autopsy, Kojima *et al*. found no significant difference in bladder wall thickness in different sections of the bladder wall. The specific gravity of the bladder wall was (0.957 ± 0.026) which was subsequently rounded to 1 in the calculations.[10] In the cadaver studies, it was found that ultrasound-estimated bladder weight correlated very well with the actual bladder weight (r = 0.97, *P* <0.0001).[10]

The reproducibility of ultrasound-estimated bladder weight at different bladder volumes was studied in 16 men at volumes ranging from 100 to 300 ml which showed a mean (%) variation of 5 g (12%).[10] Naya *et al*. reported the intra-observer and inter-observer variability among 36 patients and found no statistically significant difference.[14]

The morphometric changes in bladder outlet obstruction were described in a study of 26 patients diagnosed with BOO based on transrectal ultrasound criteria.[15] The patients underwent subcapsular prostatectomy as well as full thickness biopsies of the anterior bladder wall. These were compared to control specimens taken from the bladder wall at autopsies of men with no urinary tract disease. It is noted that the connective tissue to smooth muscle ratio was not statistically different between control bladders and BOO bladders and it correlated with ultrasound-estimated bladder weight in both groups. However the 6 cases with ultrasound-estimated bladder weight > 60 g had a connective tissue to smooth muscle ratio of more than 30% (the highest for controls was 28%) and connective tissue noticeably infiltrated into smooth muscle bundles.[16]

### Ultrasound-Estimated Bladder Weight as a Diagnostic Tool

In a study of 65 men with LUTS, ultrasound-estimated bladder weight correlated with the Abrams-Griffiths number (r = 0.478, P<0.0001) and Schafer grade of obstruction (r = 0.543, P<0.0001). Mean (SD) bladder weight in the obstructed group was 46.2 (13.3) g which was significantly higher than that in the unobstructed group [29.3 (9.4) g, *P*<0.0001].[17] Receiver operator characteristic (ROC) analysis suggested that a cut off value for ultrasound-estimated bladder weight of 35 g had 85% sensitivity and 87% specificity for diagnosis of obstruction.

A larger group of 234 patients were recruited to study the relationship between ultrasound-estimated bladder weight and other relevant variables including AUA symptom score, maximum flow rate (Q_max_), post-void residual (PVR) and presumed circle area ratio of the prostate. Multiple regression analysis showed that PVR (r = 0.490, *P*<0.0001) and presumed circle area of prostate (r = 0.468, *P*<0.0001) were significant independent determinants of ultrasound-estimated bladder weight.[18]

Guzman *et al*. presented data from 30 men with confirmed bladder outlet obstruction diagnosed using the Schafer nomogram. The results showed that ultrasound-estimated bladder weight correlated positively with the international prostate symptom score (IPSS; r = 0.710, *P* = 0.0012) and with maximum detrusor pressure (r = 0.710, *P* = 0.299) and it correlated negatively with Q_max_ (r = -0.873, *P* = 0.00001). Nevertheless there was no significant correlation between the bladder weight and the residual urine.[19]

### UEBW as a Predictor of Outcome

Kojima *et al*. studied ultrasound-estimated bladder weight in patients (males and females) with neurogenic bladder dysfunction. 25 patients were diagnosed with detrusor areflexia and underwent urodynamics as well as bladder weight measurement. This study found that ultrasound-estimated bladder weight had a negative correlation with bladder compliance (r = -0.57, *P*<0.01) and positive correlation with the degree of bladder deformity (r = 0.56, *P*<0.01) which is known to increase the risk of deterioration of the upper urinary tract.[20]

This group of researchers also studied ultrasound-estimated bladder weight as a predictor of acute urinary retention (AUR) in a group of 160 men presenting with LUTS, 31 of whom suffered AUR. Patients presenting with AUR had higher mean (SD) ultrasound-estimated bladder weight at 50.5 (15.5) g than those presenting with LUTS but with good bladder emptying [34.7 (13.6) g, *P*<0.0001]. Again using analysis of the ROC curve, a cut off value of 40 g was used to predict the presence of AUR giving a sensitivity of 74% and specificity of 71%.[21]

### UEBW as a Monitoring Tool

In a subsequent study, the group investigated the change in ultrasound-estimated bladder weight following surgical treatment of obstruction. BOO was diagnosed in a group of 33 men using a criteria based on transrectal ultrasound.[15] The findings showed that bladder weight decreased from a mean (SD) of 52.9 (22.6) g pre-operatively to 31.6 (15.8) g at 12 weeks post-prostatectomy. Interestingly in the 3 patients who started with bladder weight >80 g the post-operative bladder weight remained higher than 35 g at 12 weeks.[22]

Sironi *et al*. (2002) performed a pilot study in 32 men with LUTS suggestive of BOO investigating the effect of tamsulosin on ultrasound-estimated bladder weight using the same technique originally described by Kojima. Obstruction was diagnosed using pressure-flow studies and at baseline bladder weight in the obstructed group was significantly higher than non-obstructed and equivocal groups. The study showed a statistically significant (P<0.01) reduction in bladder weight from a mean (SD) of 64.1 (16.2) g at baseline to a mean (SD) of 55.9 (14.1) g at the first clinic visit, 30 days after commencing the alpha blocker. This decrease was maintained throughout the study.[23] Subsequently a multi-centre, double-blind, randomized, placebo controlled trial was carried out using 72 patients with LUTS suggestive of BOO and Q_ max_ less than 10 ml/s. The study reported a reduction in mean (SD) bladder weight after 12 weeks in both groups with a decrease of -4.1 (13.3) g in those allocated to placebo and -7.4 (12.7) g amongst those taking tamsulosin, however, these reductions were not statistically different (*P* = 0.281).[24] The wide SD was attributed to the large number of investigators and centers.

### UEBW in Children

Kojima *et al*. determined ultrasound-estimated bladder weight in 71 healthy children of both genders and found a linear correlation with age (r = 0.8, *P*<0.0001) which was used to create the formula: Estimated age-matched bladder weight = 0.86 × age in years + 6.9 g.[25]

However, the assumption of a spherical bladder in children was criticized by Brkljacic *et al*. who considered that it tends to underestimate bladder volume and therefore cause an underestimate in bladder weight. A 5 MHz trans-abdominal ultrasound transducer was used and the bladder shape was categorized into round, ellipsoid, cuboid or triangular. The ultrasound-estimated bladder weight was then calculated in 92 healthy children with a specific bladder volume formula for each category of bladder shape.[26] A similar correlation between bladder weight and age was found (r = 0.78, P <0.05) and a simpler formula was proposed: Bladder weight = Age + 8.4.

Kojima *et al*. also looked at ultrasound-estimated bladder weight in 82 children with various urinary disturbances and correlated the deviation of measured bladder weight from the calculated age-matched bladder weight with changes to bladder compliance. The results showed that a deviation of more than 100% from the age-matched ultrasound-estimated bladder weight predicted low compliant bladder (compliance <10ml/cmH_2_O) with a sensitivity of 80% and specificity of 97%.[25]

### UEBW Automation

**Figure 2:**
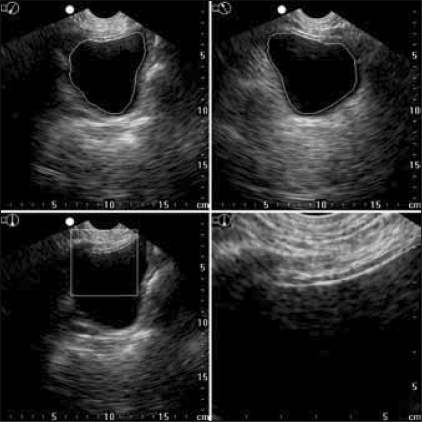
Trans-abdominal ultrasound images of the bladder at 3.7 MHz using the BVM6500 (Verathon Medical, Bothell, Washington State, USA). The software automatically identifies the bladder (white line) and the bladder wall (yellow line)

One problem with the measurement technique used in all the above studies is that bladder weight is dependent on an experienced operator measuring bladder wall thickness and all the calculations have to be done manually. The development of a handheld device with automated measurement and calculation presented a great step forward. The BladderScan^®^ BVM 6500 (Verathon Medical, Bothell, USA) acquires a V-mode ultrasound image using a 3.7 MHz transducer rotating within 120° cone.[27] The 3-dimensional ultrasound data is transferred into a computer to be analyzed using a specially developed algorithm that identifies the bladder region [Figure 2].

The inner surface of the bladder is delineated on the 3D image by the computer and the bladder surface area (SA) is calculated by triangulation. The anterior bladder wall is determined and the thickness (BWT) is measured automatically. Ultrasound-estimated bladder weight is then calculated using the formula:

UEBW = BWT × SA × specific gravity

The stated advantages of automated measurement of ultrasound-estimated bladder weight are:

1. Use of 3D rather than 2D ultrasound
2. Calculation of the actual surface area rather than assuming a spherical bladder
3. Automated and reproducible measurement

Using data from 216 scans in 20 healthy male subjects, this approach estimated mean (SD) bladder weight to be 42 (6) g.[27] It is noted that this estimate is higher than that quoted by Kojima in normal subjects of 29.3 (9.4) g.[16] This is explained by the fact that a sphere has the least surface area for a given volume and since the bladder shape is almost never absolutely a sphere, the calculation using the true surface area would produce higher estimate of bladder weight. The variation in the repeated measure of automated bladder weight measurement corresponded with a coefficient of variation of 9%.

A further study reported a mean (SD) normal range for ultrasound-estimated bladder weight measured using the BladderScan^®^ BVM 6500 of 47.8 (9.3) g in a population based group of 359 Caucasian men (age range 54-92 years) from Olmsted County, USA. The study concluded that bladder wall thickness and surface area were better correlated with symptom severity score, peak flow rate, prostate volume, and PVR when compared to manually calculated bladder weight. However, the group did not state clearly the volumes at which the scans were performed which would have a significant bearing on both bladder wall thickness and bladder surface area.[28]

The use of UEBW may not be limited to men and bladder outlet obstruction only. Spiteri *et al*. and associates submitted preliminary data in women to the International Continence Society meeting in 2006. Ultrasound-estimated bladder weight was measured using the BladderScan^®^ BVM 6500 in 25 women who underwent urodynamics for LUTS. The study found that mean (SD) bladder weight in women with detrusor overactivity [42.6 (5.8) g] was higher than that found in women with urodynamic stress incontinence [36.5 (4.9) g].[29] There are two possible causes for this difference; a reduced detrusor muscle mass due to reduced outflow resistance in stress incontinence, or an increased detrusor muscle mass in detrusor overactivity due to contractions against a closed outlet.

## METHODS (2)

We have recently studied the relationship between ultrasound-estimated bladder weight and urodynamic diagnosis in men complaining of LUTS.[30,31] Bladder weight was measured using BladderScan^®^ BVM 6500 in 34 men undergoing urodynamics for LUTS to assess its relationship with obstruction as well as detrusor overactivity (DO).

### Results (2)

The study showed that ultrasound-estimated bladder weight correlated with the Abrams-Griffiths number (r = 0.356, *P* = 0.045). It was also noted that men with DO had higher bladder weight with a mean (SD) of 53.1 (6.2) g compared to those without DO [48.9 (9.0) g; *P* = 0.03]. This raises a clinical diagnostic problem whereby increased bladder weight in men with LUTS may be due to DO alone, obstruction alone or both. Ongoing prospective clinical studies of the device are underway at both New York University Medical Center and Sant’ Andrea Hospital of “La Sapienza” university in Italy with results expected soon.[32]

## CONCLUSION

Ultrasound-estimated bladder weight is a developing non-invasive tool for investigating lower urinary tract dysfunction in both men and women. It seems to have potential as a diagnostic instrument, a clinical monitoring tool as well as a predictor of outcome. Larger multi-center studies would be of great interest.
